# Genotyping of stathmin and its association with clinical factors and survival in patients with ovarian cancer

**DOI:** 10.3892/ol.2013.1144

**Published:** 2013-01-18

**Authors:** LISHA YING, DAN SU, JIANQING ZHU, SHENGLIN MA, DIONYSSIOS KATSAROS, HERBERT YU

**Affiliations:** 1 Cancer Research Institute, Zhejiang Cancer Hospital, Hangzhou, Zhejiang, P.R. China;; 2 Department of Obstetrics and Gynecology, Gynecologic Oncology and Breast Cancer Unit, University of Turin, Turin, Italy;; 3 Cancer Epidemiology, Cancer Control and Population Sciences, Cancer Center, University of Hawaii, Hawaii, USA

**Keywords:** stathmin, genetic polymorphisms, ovarian cancer, genotype, haplotype

## Abstract

Stathmin is closely correlated with the progression and prognosis of a number of types of human cancer. The present study analyzed the associations between genetic variations in the stathmin gene and clinical outcomes of ovarian cancer. A total of 178 patients with epithelial ovarian cancer were treated with cytoreductive surgery followed by platinum-based chemotherapy. DNA was extracted from fresh tumor samples obtained during surgery. A total of 32 DNA samples were selected randomly for resequencing of the stathmin gene. Tag single nucleotide polymorphisms (SNPs) were identified based on the haplotype model as analyzed by PolyPhred software. Direct sequencing was employed in the genotyping of stathmin in 178 cases. A total of 10 nucleotide variations in stathmin were identified, of which 3 high-frequency variations were known SNPs from databases and 7 were new variations with low frequencies. The tag SNPs rs159531 and rs11376635 were selected from the linkage disequilibrium block of the gene to genotype stathmin in 178 cases. The distribution of the rs159531 genotype in ovarian cancer was 52.8% C/C, 35.4% C/T and 11.2% T/T. The distribution of the rs11376635 genotype in ovarian cancer was 32.0% G/G, 48.3% G/-, 18.5% -/-. The main haplotypes calculated by phase2.0 software were 55.6% CG, 27.8% T-, 15.4% C- and 1.2% TG. However, no associations between the stathmin genotype or haplotype and the outcomes in patients with ovarian cancer were observed. The stathmin genotype and haplotype were not associated with the phenotype of patients with ovarian cancer.

## Introduction

Epithelial ovarian cancer is the leading cause of mortality from gynecological cancer worldwide and the 5-year survival rate remains <40% despite good initial responses to standard post-operative adjuvant chemotherapy with combined paclitaxel and cisplatin (1–3). The majority of patients succumb to tumor recurrence or metastasis due to intrinsic or acquired drug resistance (4). A number of studies have demonstrated that variations in the genes for DNA repair, multi-drug resistance and drug-metabolizing enzymes were associated with the treatment response or prognosis of ovarian cancer (5–7).

Stathmin is encoded by the human STMN1 gene located at chromosome 1p36.1. The encoded protein prevents the assembly and promotes the disassembly of microtubules and is involved in the regulation of the microtubule filament system by destabilizing microtubules (8). Numerous studies have noted that upregulated expression of stathmin in several types of cancer, including breast (9), colorectal (10), endome-trial (11), head and neck (12), hepatocarcinoma (13), lung (14), ovarian (15) and prostate cancer (16), was correlated with the malignant biological behavior of cancer cells, as well as poor treatment responses to microtubule-targeting drugs (13,17,18). Our previous study also showed that high stathmin expression predicted an unfavorable prognosis in patients with ovarian cancer who received paclitaxel and platinum chemotherapy, supporting the possibility that stathmin may interfere with paclitaxel treatment, leading to a poor prognosis (19). However, few studies have reported the association between the stathmin genotype and treatment response or outcomes in patients with ovarian cancer. Moreover, the distribution, frequency and function of the variations of stathmin are not clear.

In the present study, the stathmin gene was resequenced by genotyping tag single nucleotide polymorphisms (SNPs) based on haplotype model analysis, then the associations of the stathmin genotype with treatment responses and disease progression were analyzed.

## Materials and methods

### Patients

A clinical study of ovarian cancer was conducted at the Gynecologic Oncology Unit at University of Turin (Turin, Italy) between October 1991 and February 2000. An ethics review committee at the university approved the study and all participants provided informed consent. From the study, 178 patients who had primary epithelial ovarian cancer and received post-operative platinum-based chemotherapy were identified. The median age of the patients at surgery was 57.4 years, with a range of 26 to 82 years. Of the 178 patients, 33 (18.5%) were diagnosed with stage I disease, 12 (6.7%) were stage II, 120 (67.4%) were stage III and 13 (7.3%) were stage IV. The disease staging was classified according to the criteria of the International Federation of Gynecologists and Obstetricians (FIGO) (20). The histological type determined by following the World Health Organization (WHO) criteria (21) included serous, endometrioid, mucinous, clear cell and other epithelial tumors. For the data analysis, the tumor histotypes were grouped into serous (43.3%, n=77) and non-serous (56.7%, n=101). The majority of patients (68.0%, n=121) had grade 3 tumors (poorly differentiated) and a small number had grade 2 (19.1%, n=34) and grade 1 (12.9%, n=23). When evaluated following cytoreduction, 108 (60.7%) patients had residual lesions and 70 (39.3%) patients had no residual lesions.

Following surgery, all the patients were treated with platinum-based chemotherapy; among them, 75 received platinum with paclitaxel (42.1%), while 103 received platinum without paclitaxel (57.9%). At 1 month after the chemotherapy, the treatment response was assessed based on the WHO criteria (22). A complete response (CR) required the complete disappearance of all measurable lesions, while a partial response (PR) had at least a 50% reduction in the measurable lesions. Stable disease (SD) was assigned to patients with a <50% decrease or a ≤25% increase in the size of the measurable lesions, while progressive disease (PD) was assigned when lesions increased by >25% or new lesions appeared. For non-measurable disease, the progression was defined as a doubling of CA-125 from the upper normal limit (23). For the data analysis, PR, SD and PD were grouped together as poor responders and were compared with CR. Of the 178 patients in the study, 71.9% (n=128) had a CR to treatment and 27.0% (n=48) had poor responses to treatment, which included 36 with PR, 4 with SD and 8 with PD, while 2 patients (1.1%) lacked treatment response data.

### Resequencing and haplotype construction of stathmin

Genomic DNA was extracted from tumor samples following manual homogenization using the QIAamp1 DNA Mini kit (Qiagen, Valencia, CA, USA). A total of 32 DNA samples were randomly selected for resequencing from 1 kbp upstream to 1 kbp downstream of the stathmin gene. The entire coding exon and the flanking intronic sequences of the stathmin gene, covering 8.7 kbp, were amplified by PCR using the 16 pairs of primers designed by the online Primer3 software (http://primer3.wi.mit.edu/). The primer sequences for PCR are shown in Table I. A 20-*μ*l mixture was prepared for each reaction and included 1X HotStarTaq buffer, 2.0 mM Mg^2+^, 0.2 mM dNTP, 0.2 *μ*M of each primer, 1 unit HotStarTaq polymerase (Qiagen) and 1 *μ*l template DNA (5–10 ng/*μ*l). The cycling program was 95°C for 15 min; 11 cycles of 94°C for 15 sec, 62-0.5°C per cycle for 40 sec, 72°C for 1 min; 24 cycles of 94°C for 15 sec, 56°C for 30 sec, 72°C for 1 min; and 72°C for 2 min. The PCR products were purified with shrimp alkaline phosphatase enzyme (SAP; Promega, Madison, WI, USA) and exonuclease I (Exo I; Epicentre, Madison, WI, USA). Cycle sequencing was performed with the BigDye Terminator Cycle Sequencing Ready Reaction kit (Applied Biosystems, Foster City, CA, USA) on an ABI 3130 Genetic Analyzer (Applied Biosystems). The primer sequences for sequencing are shown in Table II. SNPs were identified by the PolyPhred program (http://droog.mbt.washington.edu/poly_get.html). Pairwise linkage disequilibrium (LD) between the sequence variations was analyzed by two parameters, |D’| and r^2^. The haplotypes from the sequence variations were reconstructed with the PHASE 2.1 program (http://www.stat.washington.edu/stephens/phase/download.html).

### SNP genotyping in 178 ovarian cancer tissue samples

Direct sequencing in 178 patients with ovarian cancer was used to genotype 2 tag SNPs with high allelic frequency identified according to the haplotype model. The method of sequencing was as mentioned previously.

### Statistical analysis

The frequencies of various genotypes and haplotypes were compared between patients with and without treatment responses using the Chi-square test. Multivariate analyses were performed with the use of unconditional logistic regression analysis to assess the associations between the treatment responses and each genetic polymorphism while adjusting for patient age at diagnosis, tumor histology, disease stage, grade and residual tumor. The odds ratios (ORs) and corresponding 95% confidence intervals (CIs) were calculated using the logistic regression model. By combining the data of stathmin expression from our previous study (19), the associations between the phenotype and genotype of stathmin were analyzed using Pearson’s correlation test. All P-values reported were two-sided and P≤0.05 was considered to indicate statistically significant differences. The statistical analysis was performed using SPSS software, Version 11.0 (SPSS Inc., Chicago, IL, USA).

## Results

### Variations in the stathmin gene

As shown in Table III, 10 variations in stathmin were identified, including 2 insertion/deletion and 8 single nucleotide variations, of which 3 high-frequency variations were known SNPs from the dbSNP database of the NCBI website, rs213641 (G/T) in intron 1, rs159531 (T/C) in intron 3 and rs11376635 (-/G) separately in the 3’UTR. The distributions of these SNPs in 32 samples were similar to the data from the dbSNP database. Of the variations, 7 were reported for the first time and all were present at low frequencies.

### Tag SNP and haplotype in stathmin

The analysis of pairwise LD and r^2^ showed these 10 variation loci were in high LD and the entire gene was in an LD block. Based on parameters including r^2^>0.8 and allelic frequency >10%, 2 tag SNPs were identified, rs159531 (T/C) and rs11376635 (-/G). According to the 2 tag SNPs, the main haplotypes were calculated as CG, T- and C-.

### Genotyping of stathmin in 178 patients with ovarian cancer

Using direct sequencing in 178 patients with ovarian cancer, 2 tag SNPs with high allelic frequency, rs159531 (G/T) and rs11376635 (T/C), were identified for genotyping. The genotypes of rs159531 were 52.8% C/C (94/178), 35.4% C/T (63/178), 11.2% T/T (20/178) and 0.6% missing (1/178). The genotypes of rs11376635 were 32% G/G (57/178), 48.3% G/- (86/178), 18.5% -/- (33/178) and 1.1% missing (2/178). The distribution of the main haplotypes were 79.8% CG (142/178), 9.6% T- (17/178), 9.0% C- (16/178) and 1.7% TG (3/178; Table IV).

### Correlation of the genotypes of stathmin and clinical characteristics of ovarian cancer

As shown in Table V, the genotypes or haplotypes of stathmin were not observed to be associated with disease stage, tumor grade, histology, residual tumor size or treatment response in 178 patients in ovarian cancer.

No significant differences in the survival of patients with ovarian cancer according to the genotypes of stathmin were observed.

### Association of genotype and phenotype in stathmin

By combining the present data with our previous mRNA expression of stathmin data, no significant correlations between the genotype and phenotype of stathmin were observed, as shown in Table VI.

## Discussion

Stathmin, first identified as a cytosolic phosphoprotein in neuroendocrine cells (24), has been observed to be closely correlated with the progression and prognosis of a number of types of human cancer. High expression levels of stathmin indicate increased proliferation, invasion and poor prognosis (25). However, few studies have reported the somatic or genetic variants of the stathmin gene. In the present study, 10 nucleotide variations of stathmin, including 3 SNP sites with high frequency from the dbSNP database of the NCBI website and 7 new variations with low frequencies, were identified by resequencing. All were non-coding variations, 2 located in 3′UTR and the others in introns. Variations in the 3′UTR may have significant functional implications for miRNA binding and posttranscriptional regulation and be associated with human diseases (26,27). Additionally, increasing evidence indicates that genomic variants in non-coding sequences may have unexpected deleterious effects on the splicing of the gene transcript (28).

In order to observe the possible biological function of those variations, 2 tag SNPs with high allelic frequency (rs159531 and rs11376635) were selected for genotyping and the associations between the genetic variants of stathmin and clinical characteristics of 178 patients with ovarian cancer were analyzed. The genotypic frequencies of these 2 tag SNPs were similar to the data from the dbSNP database. The majority of patients (79.8%, 142/178) had the CG haplotype. However, the genotype of the tag SNP or haplotype was not associated with the expression level of stathmin and no significant associations among genotype, clinical characteristics and outcomes in patients with ovarian cancer were observed.

Stathmin is a microtubule-destabilizing protein that regulates microtubule dynamics by preventing tubulin polymerization and promoting microtubule disassembly during cell cycle progression (29,30). Changes to the mRNA levels of the stathmin gene may disturb microtubule stabilization and thereby affect the treatment response in cancer therapy, particularly in microtubule-targeting drugs. A large number of studies have reported that the overexpression of stathmin may be an independent predictor of poor treatment responses or worse prognoses and a potential target in numerous types of cancer (31–34). We hypothesized that genetic variation in stathmin is associated with upregulated expression of stathmin which may affect the outcome of patients with ovarian cancer. However, no significant associations were observed in the present study. To the best of our knowledge, no study has shown germline or somatic variations of stathmin in cancer, although certain studies have reported that SNPs in stathmin were associated with the etiopathogenesis of a broad range of neuropsychiatric disorders with dysfunctional networking. For example, SNP rs182455, located in the promoter of stathmin and rs213641, an SNP in the 5′UTR of the alternatively transcribed exon 1c in stathmin, which may modify the binding of nerve growth factor-induced protein C, were identified in fear and anxiety processing and cognitive and affective control processes (35,36). By contrast, Buttmann *et al* also analyzed rs182455 in 647 clinically well-characterized multiple sclerosis (MS) patients and 519 healthy controls, but no associations of the genotype of rs182455 SNPs with MS susceptibility or clinical disease course were observed (3). Furthermore, rs12037513 and rs159522, both located in close vicinity to the stathmin gene, have no any association with schizophrenia (37).

In conclusion, although no significant associations were observed between the genotype of stathmin and clinical characteristics or outcomes in patients with ovarian cancer in the present study, the results provided information concerning somatic or genetic variations of stathmin in patients with ovarian cancer.

## Figures and Tables

**Table I:** Sathmin primer sequences for PCR.

No.	Forward primer	Reverse primer
1	AATGCCGCAACAAGCATATTT	CAGAGCAGCACTGGGTTCTTT
2	CAATTTCCTTTGTGCCTTTGC	GGTCCTTCCCTACCTTTCCAA
3	ACTGCTCTGTCCGAGTGCTG	CCCGAGCCACACACAAAG
4	GCTGAGGCCAGCAAGAGG	GGTCCAATCCGGGTAACTCC
5	GAACTGTGAAGGGGGTGGTG	GTCTGTGTCTGACGTGGTGGA
6	TGTTGGGCAAGGAAGCTTAAA	CAAATCAAAGGCGAAGACCTG
7	TTCACCATGGCTTCTTCTGGT	GGGCTGATGAGGAAAGTTGTG
8	TTGCCTGCAAATACATCTTCC	GGCAACACCATGTATTAAAGGAGA
9	TGGAAGGAAATACCAGTCCTCA	TGTAAGCACTGAGGCTCTTCG
10	TGATTGTGTTGCTCAGCTGGT	TTGCGAGTGGCACTTTTATTG
11	TTTACAATGAGCTAGTTTTCTTTGG	CAGCTTCATGGGACTGGAAAA
12	CCACACCCAGCCTGAATACAT	CAGTCTCGTCAGCAGGGTCTT
13	GTTGTGTTGGGCCTCTTTGAG	GAGGGGCTCTATGGCTTGATT
14	CAATCCCAATTCTGTCCCAAT	TGAGAGGCAAAGCACTGACAA
15	TGACTCGGGTGGTTAAGGTTG	TATTTGCCCTACATGGGCGTA
16	TTCAACCAGAGGCTAATGAGTGA	ATGCATCCCCTTCAGTTTCCT

**Table II:** Sathmin primer sequences for sequencing.

No.	Sequencing primer
1	AATGCCGCAACAAGCATATTT
2	GGTCCTTCCCTACCTTTCCAA
3	CCCGAGCCACACACAAAG
4	GGTCCAATCCGGGTAACTCC
5	GTCTGTGTCTGACGTGGTGGA
6	CAAATCAAAGGCGAAGACCTG
7	TTCACCATGGCTTCTTCTGGT
8	GGCAACACCATGTATTAAAGGAGA
9	TGTAAGCACTGAGGCTCTTCG
10	TTGCGAGTGGCACTTTTATTG
11	CAGCTTCATGGGACTGGAAAA
12	CCACACCCAGCCTGAATACAT
13	GAGGGGCTCTATGGCTTGATT
14	TGAGAGGCAAAGCACTGACAA
15	TATTTGCCCTACATGGGCGTA
16	ATGCATCCCCTTCAGTTTCCT

**Table III:** Information of 10 DNA variations of stathmin.

Name	Position in NC_000001.9	Reference allele (A1)	Other allele (A2)	A2 (%)	Variation property	Nucleotide variation
Variation 1	26105623	C	T	3.1	INTRON1	C/T
Variation 2 (rs213641)	26104943	G	T	56.3	INTRON1	G/T
Variation 3	26103346	T	C	6.2	INTRON2	T/C
Variation 4	26102107	C	T	3.1	INTRON3	C/T
Variation 5 (rs159531)	26100996	T	C	68.8	INTRON3	T/C
Variation 6	26100558	T	C	6.2	INTRON4	T/C
Variation 7	26100349^26100360	N	GG	3.1	INTRON4	N/GG^a^
Variation 8	26100236	G	T	3.1	INTRON4	G/T
Variation 9	26098752	A	T	3.1	3’UTR	A/T
Variation 10 (rs11376635)	26098388^26098389	-	G	56.3	3’UTR	-/G

a N=tgttaggttct.

**Table IV:** Patient clinicopathological characteristics and geno-types.

Variable	Value
Age (years), median (range)	57. 4 (26–82)
Stage, n (%)	
I–II	45 (25.3)
III–IV	133 (74.7)
Grade, n (%)	
1–2	57 (32.0)
3	121 (68.0)
Histology, n (%)	
Non-serous	101 (56.7)
Serous	77 (43.3)
Residual tumor, n (%)	
No residual	70 (39.3 )
Residual	108 (60.7)
Treatment response, n (%)	
Complete response	128 (71.9)
Poor response	48 (27.0)
Missing	2 (1.1)
Chemotherapy, n (%)	
Platinum with paclitaxel	75 (42.1)
Platinum without paclitaxel	103 (57.9)
rs159531, n (%)	
C/C	94 (52.8)
C/T	63 (35.4)
T/T	20 (11.2)
Missing	1 (0.6)
rs11376635, n (%)	
G/G	57 (32.0)
G/-	86 (48.3)
-/-	33 (18.5)
Missing	2 (1.1)
Haplotype, n (%)	
C/G	142 (79.8)
T/-	17 (9.6)
C/-	16 (9)
T/G	3 (1.7)

**Table V:** Correlation of the genotype of stathmin with the clinical characteristics of ovarian cancer.

Variable	rs159531, n (%)			rs11376635, n (%)			Haplotype, n (%)	
		
	C/C	C/T	T/T	G/G	G/-	-/-	C/G	T/-, C/-, T/G
Stage								
I–II	24 (25.5)	18 (28.6)	3 (15.0)	15 (26.3)	20 (23.3)	10 (30.3)	35 (24.6)	10 (27.8)
III–IV	70 (74.5)	45 (71.4)	17 (85.0)	42 (73.7)	66 (76.7)	23 (69.7)	107 (75.4)	26 (72.2)
P-value	0.478	-	-	0.724	-	-	0.674	-
Grade								
1–2	32 (34.0)	17 (27.0)	8 (40.0)	19 (33.3)	26 (30.2)	12 (36.4)	45 (31.7)	12 (33.3)
3	62 (66.0)	46 (73.0)	12 (60.0)	38 (66.7)	60 (69.8)	21 (63.6)	97 (68.3)	24 (66.7)
P-value	0.475	-	-	0.801	-	-	0.844	-
Histology								
Non-Serous	57 (60.0)	32 (50.8)	11 (55.0)	32 (56.1)	47 (54.7)	20 (60.6)	80 (56.3)	21 (58.3)
Serous	37 (39.4)	31 (49.2)	9 (45.0)	25 (43.9)	39 (45.3)	13 (39.4)	62 (43.7)	15 (41.7)
P-value	0.47	-	-	0.842	-	-	0.853	-
Residual tumor								
No residual	41 (43.6)	24 (38.1)	5 (25.0)	28 (49.1)	31 (36.0)	11 (33.3)	59 (41.5)	11 (30.6)
Residual	53 (56.4)	39 (61.9)	15 (75.0)	29 (50.9)	55 (64.0)	22 (66.7)	83 (58.5)	25 (69.4)
P-value	0.29	-	-	0.207	-	-	0.256	-
Treatment response								
Complete responder	67 (72.8)	45 (71.4)	15 (75.0)	40 (72.7)	63 (73.3)	23 (69.7)	102 (72.9)	26 (72.2)
Poor responder	25 (27.2)	18 (28.6)	5 (25.0)	15 (27.3)	23 (26.7)	10 (30.3)	38 (27.1)	10 (27.8)
P-value	0.95	-	-	0.925	-	-	1.000	-

**Table VI:** Correlation of genotype, haplotype and stathmin expression.

	Stathmin expression (%)			
	Low	Median	High	P-value
rs159531				
C/C	33 (35.1)	29 (30.9)	32 (34.0)	
C/T	19 (30.2)	22 (34.9)	22 (34.9)	0.960
T/T	7 (35.0)	7 (35.0)	6 (30.0)	
rs11376635				
G/G	22 (38.6)	16 (28.1)	19 (33.3)	
G/-	23 (26.7)	30 (34.9)	33 (38.4)	0.366
-/-	14 (42.4)	11 (33.3)	8 (24.2)	
Haplotype				
C/G	44 (31.0)	46 (32.4)	52 (36.6)	0.345
T/-, C/-, T/G	15 (41.7)	12 (33.3)	9 (25.0)	
